# Analysis of Microbial Community, Volatile Flavor Compounds, and Flavor of Cigar Tobacco Leaves From Different Regions

**DOI:** 10.3389/fmicb.2022.907270

**Published:** 2022-06-10

**Authors:** Tianfei Zheng, Qianying Zhang, Pinhe Li, Xinying Wu, Yi Liu, Zhen Yang, Dongliang Li, Juan Zhang, Guocheng Du

**Affiliations:** ^1^School of Biotechnology, Jiangnan University, Wuxi, China; ^2^Key Laboratory of Industrial Biotechnology, Ministry of Education, School of Biotechnology, Jiangnan University, Wuxi, China; ^3^Science Center for Future Foods, Jiangnan University, Wuxi, China; ^4^Cigar Fermentation Technology Key Laboratory of China Tobacco, China Tobacco Sichuan Industrial Co., Ltd., Chengdu, China

**Keywords:** cigar tobacco leaves, microbial community, sensory analysis, flavor, volatile flavor compounds

## Abstract

Despite the booming international trade in cigar tobacco leaves (CTLs), the main characteristics of tobacco leaves from different producing areas are rarely reported. This study aimed to characterize the microbial community, volatile flavor compounds (VFCs), and flavor of CTLs from four famous cigar-producing areas, including Dominica, Brazil, Indonesia, and China. High-throughput sequencing results showed that the dominant genera in CTLs were *Staphylococcus, Pseudomonas, Aspergillus, Sampaiozyma*, and *Alternaria*. Sensory analysis revealed that Indonesian and Chinese CTLs were characterized by leathery, peppery, and baked aroma. Brazilian CTLs were dominated by caramel and herb aroma. Dominican CTLs had aromas of milk, fruity, sour, cream, flower, nutty, and honey. Supplemented with the determination of volatile flavor compounds (VFCs), the flavor of CTLs could be scientifically quantified. Most of these VFCs were aldehydes and ketones, and 20 VFCs showed significant differences in CTLs from different regions. The microbial community, VFCs, and flavor of CTLs vary widely due to geographic differences. Network analysis revealed the microbial community was closely related to most VFCs, but the relationships between the fungal community and VFCs were less than the bacterial community, and most of them were negative. Furthermore, it also found that the bacterial community had a greater contribution to the flavor of CTLs than the fungal community. This study obtained essential information on CTLs, which laid a foundation for deeply excavating the relationship between microbes and VFCs and flavor, and establishing a tobacco information database.

## Introduction

Cigar, a kind of tobacco product rolled from dried and fermented cigar tobacco leaves (CTLs), is famous worldwide because of its profound cultural heritage and incredible taste (Viola et al., 2016; Allem et al., 2019). Compared with flue-cured tobacco, cigars have a more mellow and varied flavor, usually containing aromas of fruit, nuts, coffee, milk, and cedar (Morris and Fiala, 2015). The flavor of CTLs is closely related to the natural environment and fermentation technology of the producing area (Xia et al., 2014; Yin et al., 2019). The cultivation of CTLs requires suitable environmental conditions. Only some places worldwide that can produce high-quality CTLs, such as Cuba, Brazil, Cameroon, Dominica, Honduras, Indonesia, Mexico, Nicaragua, America, China, and Southeast Asia (Zhang et al., 2021a). Their special climate (temperature, sun exposure time, and rainfall) and soil environment create the inimitable flavor of CTLs from different geographical regions (Zhang et al., 2013). For example, Cuban CTLs have a strong spicy flavor; Dominican CTLs are known for their smoothness and gentleness; Chinese CTLs have a light and mellow aroma (Stubbs, 2010). Additionally, CTLs need to be fermented by the microbial community in CTLs to become usable. The metabolic activities of a microbial community, including degradation of carbohydrates, degradation of chlorogenic acid, degradation of proteins, Strecker degradation, and caramelization reactions, fatty acid and lipid biosynthesis, amino acid biosynthesis, and aromatic compound biosynthesis, have important contributions to tobacco aroma formation (Banozic et al., 2020). Differences in structure and function of the microbial community also lead to differences of tobacco flavor (Yang et al., 2021). However, the flavor and microbial community of CTLs from different regions have not been fully reported and the relationship between flavor and microbes requires further study.

The development of omics technologies such as 16S rRNA gene, shotgun sequencing, and metabolomics have provided feasible solutions to identify species, genes, proteins, and metabolites in native ecosystems (Zhang et al., 2021b; Romdhane et al., 2022). These technologies have also been applied to tobacco samples. For example, smokeless tobacco has been found to be dominated by phyla *Firmicutes, Proteobacteria, Actinobacteria*, and *Bacteroidetes* (Han et al., 2016). In addition, many studies have shown that cigarette tobacco was dominated by the genera *Bacillus* and *Pseudomonas* (Su et al., 2011; Ye et al., 2017). Xia et al. systematically investigated the metabolic profiling of tobacco leaves from different geographical origins (Xia et al., 2014). They screened some important metabolites related to the planting regions and climate factors. These results indicated that the planting environment has a more significant effect on metabolic changes than genetics. Moreover, the flavor of CTLs is generally evaluated by professional tasters. However, sensory evaluation is easy to be influenced by human factors, and sometimes it is not so objective. Therefore, sensory evaluation should be supplemented with knowledge of flavor compounds.

This study used high-throughput sequencing, sensory evaluation, and metabolomics to characterize the microbial community, flavor, and volatile flavor compounds (VFCs) of CTLs from four famous cigar-producing regions, including Dominica, Brazil, Indonesia, and China. Differences in flavor, VFCs, and microbial communities of CTLs in different regions were investigated, and their relationships were analyzed. These results may provide a scientific basis and guidance for the evaluation and regulation of CTLs.

## Materials and Methods

### Cigar Tobacco Leaves Collection

A total of 24 CTLs from four well-known cigar production areas, including 6 CTLs from Dominica, 8 CTLs from Indonesia, 6 CTLs from Brazil, and 4 CTLs from China (Table 1), were collected by China Tobacco Sichuan Industrial Co., Ltd., the CTLs were randomly sampled the four corners and center of the tobacco stack, 500–1,000 g each sample, then CTLs were mixed evenly and put into sterile bags for sealing. At the same time, the sample information, sampling time, and sampling place were marked, and then store at −30°C until detection.

**Table 1:** Information of cigar tobacco leaves.

**Place**	**Sample**	**harvesting time**	**Latitude**	**Climate type**	**Collection time**
Brazil	BX	2016	13°26′S	Tropical dry and wet season climate	2020
	BXH	2016			2020
	BaF	2016			2020
	BaFH	2016			2020
	BaM	2009			2020
	BaMH	2009			2020
China	D3	2017	31°13′N	Subtropical monsoon climate	2020
	D3H	2017			2020
	GW3	2010			2020
	GW3H	2010			2020
Dominica	DC	2015	18°48′N	Tropical rainforest climate	2020
	DCH	2015			2020
	DN	2015			2020
	DNH	2015			2020
	DSC	2015			2020
	DSCH	2015			2020
Indonesia	C	2010	6°19′S	Tropical rainforest climate	2020
	D	2010			2020
	A	2010			2020
	B	2010			2020
	E	2013			2020
	F	2013			2020
	YN	2010			2020
	YNH	2010			2020

### Microbial Community Analysis

To collect microbes from the CTLs, ~5 g CTLs were added to a 250-ml flask containing 100 ml filtered normal saline (0.9% NaCl, pH 7) and oscillated at 4°C, 220 rpm for 4 h. CTLs were removed by gauze, and microbes were collected by centrifuging at 7,000 × *g* for 15 min. Total microbial genomic DNA was extracted from the microbial cells using the DNeasy PowerSoil Kit (QIAGEN, Inc., Venlo, Netherlands), following the manufacturer's instructions. All extracted DNA samples were stored at −20°C until further analysis, and the quality of the extracted DNA was evaluated using agarose gel electrophoresis.

The bacterial and archaeal 16S rRNA genes V4–V5 region was performed using the forward primer 515F (Parada et al., 2016) (5′-GTGCCAGCMGCCGCGGTAA-3′) and the reverse primer 907R (5′-CCGTCAATTCMTTTRAGTTT-3′). The fungal internal transcribed spacer gene was amplified using the universal primers ITS1F (5′-CTTGGTCATTTAGAGGAAGTAA-3′) and ITS2R (Taylor et al., 2016) (5′-GCTGCGTTCTTCATCGATGC-3′). Each 25-μl PCR volume contained 5 μl Q5 reaction buffer (5×), 5 μl Q5 High-Fidelity GC buffer (5×), 0.25 μl Q5 High-Fidelity DNA Polymerase (5 U/μl, NEB), 2 μl (2.5 mM) dNTPs, 1 μl forward primer (10 μM; final: 0.4 μM), 1 μl reverse primer (10 μM; final: 0.4 μM), 2 μl DNA template, and 8.75 μl nuclease-free water. Amplification was achieved using the following thermocycler conditions: initial denaturation at 98°C for 2 min, followed by 28 cycles of denaturation at 98°C for 15 s, annealing at 55°C for 30 s, and extension at 72°C for 30 s. The final extension was performed at 72°C for 5 min. Amplified PCR products were purified using Agencourt AMPure Beads (Beckman Coulter, Inc., Brea, CA, USA), quantified using the PicoGreen dsDNA Assay Kit (Invitrogen, Carlsbad, CA, USA), pooled in equal amounts, and subjected to paired-end 2×300 bp sequencing using the MiSeq platform and MiSeq Reagent Kit v3 (Illumina, San Diego, CA, USA).

The gene sequences were processed using QIIME 2 (Bolyen et al., 2019). Briefly, raw sequencing reads were assigned to specific samples using exact matches to barcode sequences, and filtering was performed to exclude low-quality sequences, which were defined as those with lengths of <150 bp, average Phred scores of <20, ambiguous bases, and/or mononucleotide repeats of >8 bp. The remaining high-quality paired-end reads were assembled using FLASH (Magoc and Salzberg, 2011). After chimera detection and removal, the remaining high-quality sequences were clustered into amplicon sequence variants (ASVs). Taxonomic classification was performed using the q2-feature-classifier QIIME 2 plugin to implement the classify-sklearn method (Pedregosa et al., 2011) and the pre-trained SILVA database (version 132) (Quast et al., 2013), with 99% similarity.

### Volatile Flavor Compounds Analysis

Volatile flavor compounds (VCs) in CTLs were analyzed by headspace solid phase microextraction-gas chromatography–mass spectrometry (HS-SPME-GC-MS). CTLs were dried at 40°C and pulverized by a grinder. A total of 1.5 g powder was placed in a 10 ml glass vial and extracted by headspace solid-phase microextraction (50/30 μm DVB/CAR/PDMS fiber, Supelco, Bellefonte, PA, USA) at 60°C for 30 min. After extraction, volatile flavor compounds (VCFs) were identified using a Pegasus BT GC-TOFMS (LECO Co., St. Joseph, MI, USA), with a DB-5MS column (60 m × 0.25 mm id × 0.25 μm film thickness). Helium C-60 was used as a carrier gas with a flow rate of 1 ml/min, and the injector port was heated to 250°C. The oven temperature was fixed at 40°C for 2 min, increased to 250°C at a rate of 10°C/min, and then held for 5 min. Meanwhile, the transfer line and ion source temperatures were maintained at 280°C and 210°C, respectively. Electron impact (EI) was used as the ionization mode, with an EI voltage of 70 eV, and a mass scan range of 33–400 m/z was used for full-scan mode with an acquisition rate of 10 scans/s. Peak identification was accomplished by comparing the sample MS spectra to those of chemical standards (when available), the National Institute of Standards and Technology spectral library (NIST 14, https://www.nist.gov), and experimental and theoretical Kovats index values (Babushok and Linstrom, 2004).

### Sensory Analyses

According to a standardized procedure, the quality score of cigars was blindly assessed by a tasting panel consisting of eight professional tasters. With 10–20 years of testing experience, these tobacco tasters have conducted a sensory evaluation on more than 2,000 cigar samples, and can accurately, consistently, and repeatedly evaluate cigars. During the sensory session, a total of 20 descriptive terms for the cigars based on the Wine Aroma Wheel proposed by Noble, A. C were used to evaluate CTLs (Noble et al., 1984), including nutty, bean, woody, peppery, fruity, freshness, caramel, honey, sweet, flowery, herb, milky, cream, resin, baked, earth, hay, leathery, sour, and rouge. Different flavor characteristics were scored from 0 to 9.

### Statistical Analysis

R v. 4.0.0 was used to generate the heatmap and performed principal component analysis (PCA), boxplot analysis, and multiple comparisons. Principal component analysis (PCA) and partial least squares regression (PLSR) analysis were used to explore the relationship between VFCs and the flavor characteristics of CTLs through SMICA 14.1 (Umetrics, Umeå, Sweden). The Galaxy (https://huttenhower.sph.harvard.edu/galaxy/) was used for LEfSe analysis to assess significant differences of CTLs from different regions. Additionally, the correlation between the representative microbes (The top 40 bacterial and fungal genera) and core VFCs based on Spearman's correlation coefficients (*p* < 0.05, |*r*|>0.3), network analysis was performed by using Gephi software. All data have been standardized during the statistical analysis.

## Results

### Overview of Microbial Community

**Figure 1:**
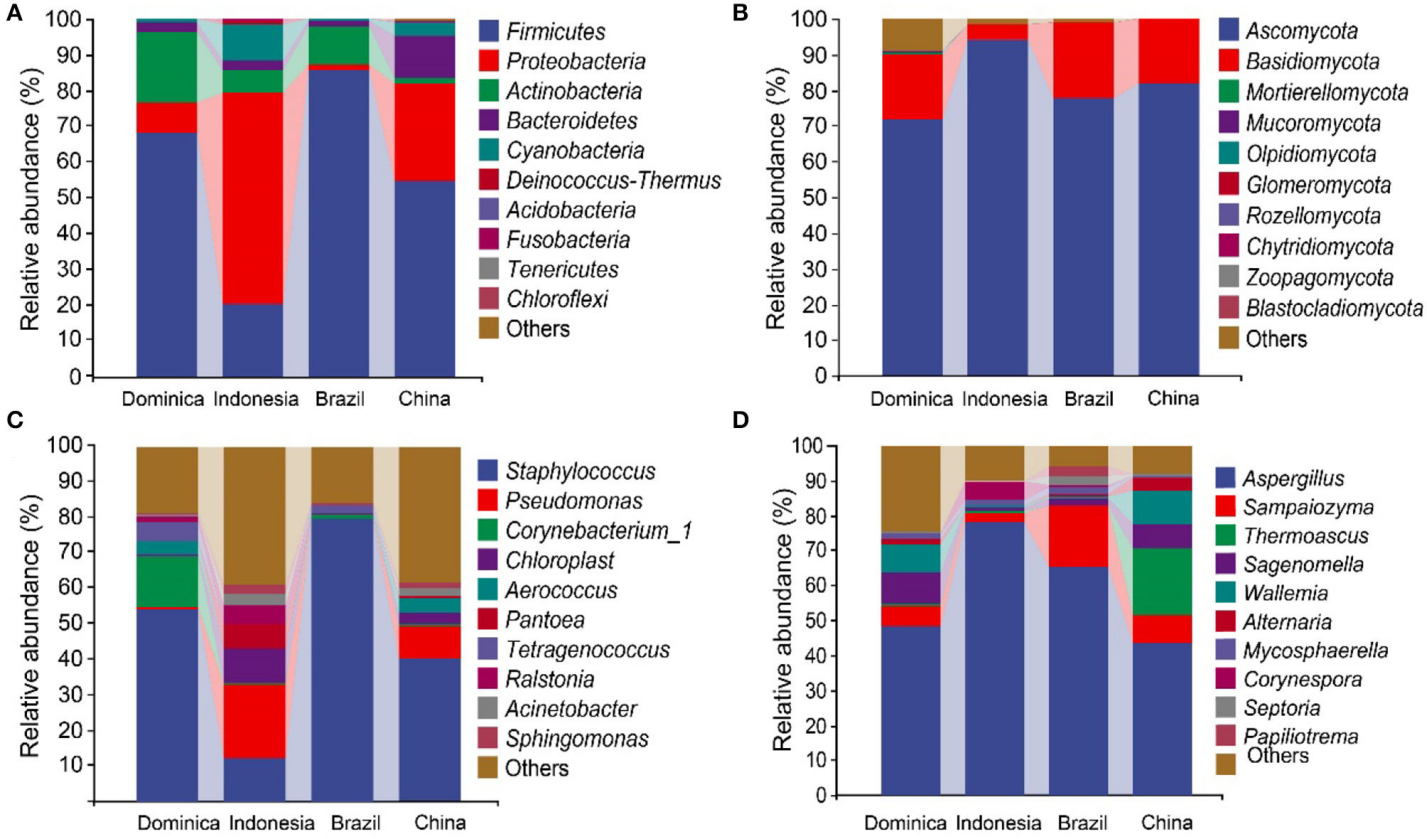
Microbial communities in cigar tobacco leaves. The top 10 predominant bacterial phyla **(A)** and genera **(C)**. The top 10 predominant fungal phyla **(B)** and genera **(D)**.

The high-throughput sequencing generated 3,011,160 high-quality reads bacterial 16S rRNA V4–V5 sequences from 24 samples, ranging from 18,813 to 71,520 reads per sample. We also obtained 2,979,634 high-quality reads from fungal ITS1 sequences, ranging from 26,634 to 61,475 reads per sample. Taxonomic analysis of the reads revealed that *Firmicutes, Proteobacteria, Actinobacteria*, and *Ascomycota* were dominant at the phylum level (Figures 1A,B). The dominant groups of bacterial genera were *Staphylococcus, Pseudomonas, Sphingomonas, Aerococcus*, and *Chloroplast* (Figure 1C), meanwhile, the dominant groups of fungal genera were *Aspergillus, Sampaiozyma, Alternaria, Alternaria*, and *Thermoascus* (Figure 1D). However, the microbial abundances varied significantly in the different regions. The relative abundances of phyla *Proteobacteria* were significantly higher in the CTLs from Indonesia and China than those in Brazil and Dominica. In contrast, *Firmicutes* were abundant in the CTLs from Brazil and Dominica than in China and Indonesia. At the genera level, the difference in microbial abundance in CTLs from different regions was more significant, such as *Staphylococcus* (Brazil 79.66%, Indonesia 12.56%), *Pseudomonas* (Indonesia 20.27%, Brazil 0.38%), *Aspergillus* (Indonesia 78.31%, China 43.66%), and *Sampaiozyma* (Brazil 17.67%, Indonesia 2.40%).

**Figure 2:**
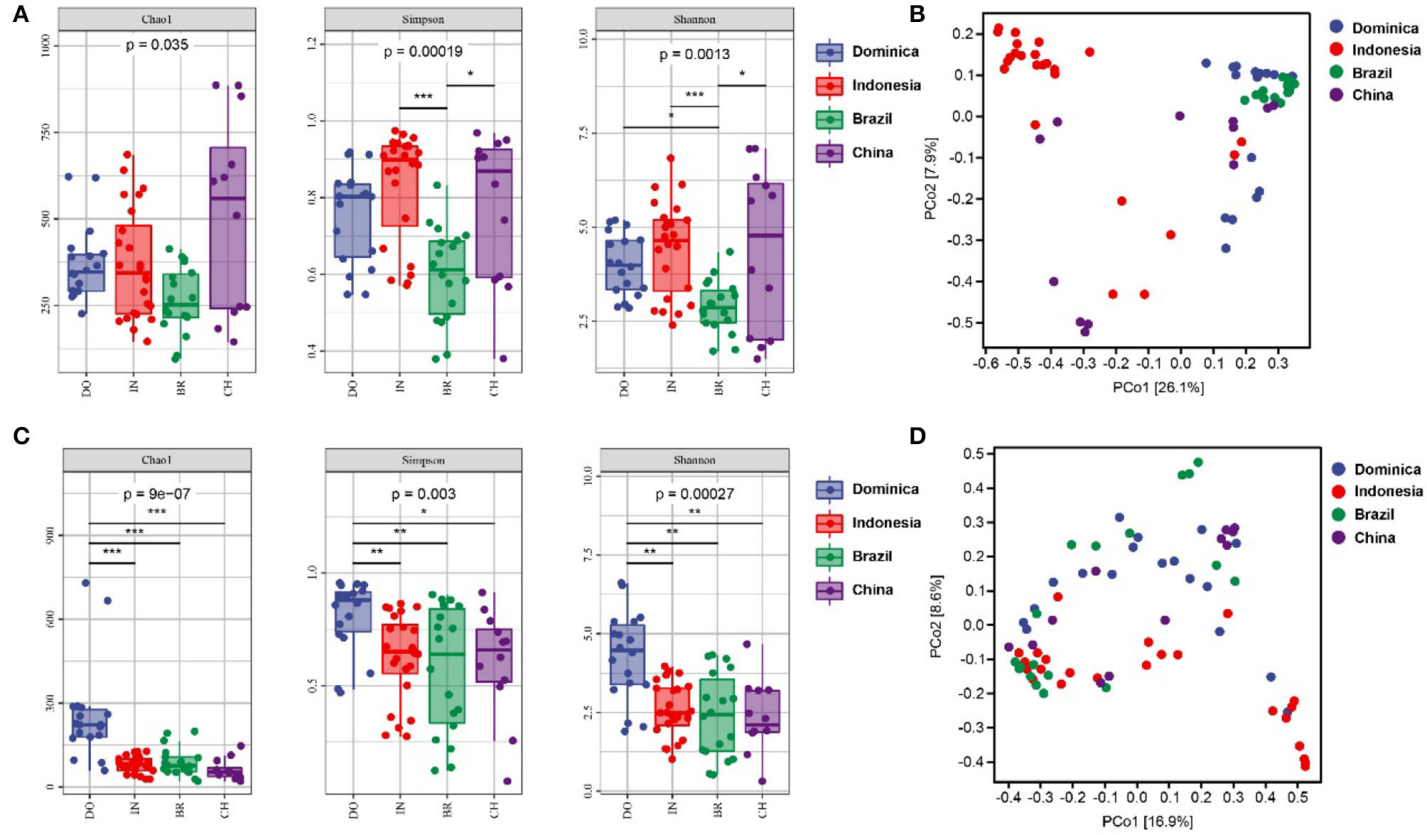
Bacterial alpha diversity **(A)** and fungal alpha diversity **(C)** were determined based on the Chao1 index, the Shannon index, and the Simpson index. Bacterial beta diversity **(B)** and fungal alpha diversity **(D)** was measured by bray_curtis distance. * < 0.05; ** < 0.01; *** < 0.001.

Microbial diversities were analyzed to explore the difference of microbial communities in CTLs from different regions. For alpha diversity, the richness and evenness of bacterial community in Indonesian and Chinese CTLs were higher than those in Dominica and Brazil (Figure 2). The richness and evenness of the fungal community in Dominican CTLs were significantly higher than that in Indonesia, Brazil, and China. When considering microbial beta-diversity based on the abundance-related Bray-Curtis distance, we found that the geographical position explained 26.1% of the bacterial variance (Figure 2B) and 16.9% of the fungal variance (Figure 2D). In all, we found a strong geographical effect upon both microbial alpha and beta diversity estimates.

**Figure 3:**
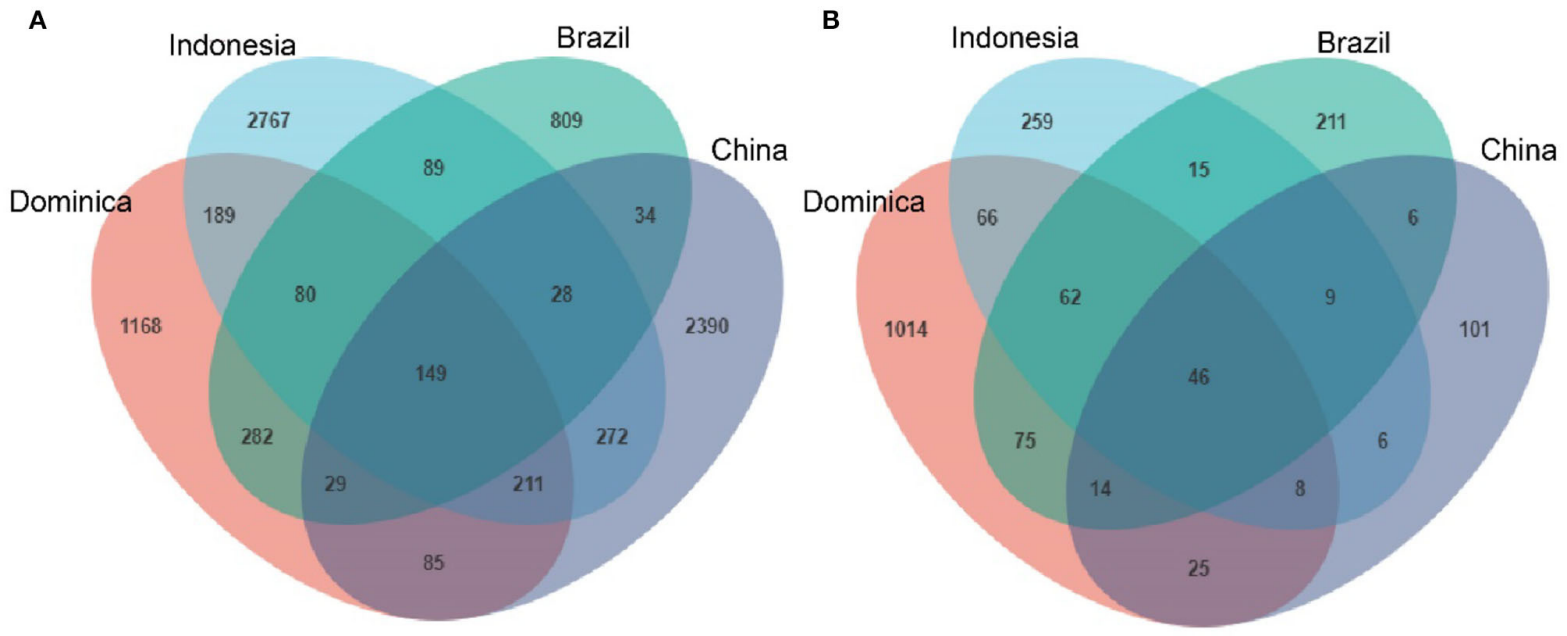
Numbers of unique and shared bacterial **(A)** and fungal **(B)** microbiota in cigar tobacco from different regions.

We then evaluated shared and unique microbes in CTLs across different regions using Venn diagrams to better visualize the overlap of microbial communities. As shown in Figure 3, there are 149 shared-bacterial community memberships (Figure 3A) and 46 shared-fungal community memberships (Figure 3B). The number of shared microbes in CTLs from all four regions was far lower than the unique microbes of each region. It could be concluded that CTLs in different regions harbored different microbial communities.

**Figure 4:**
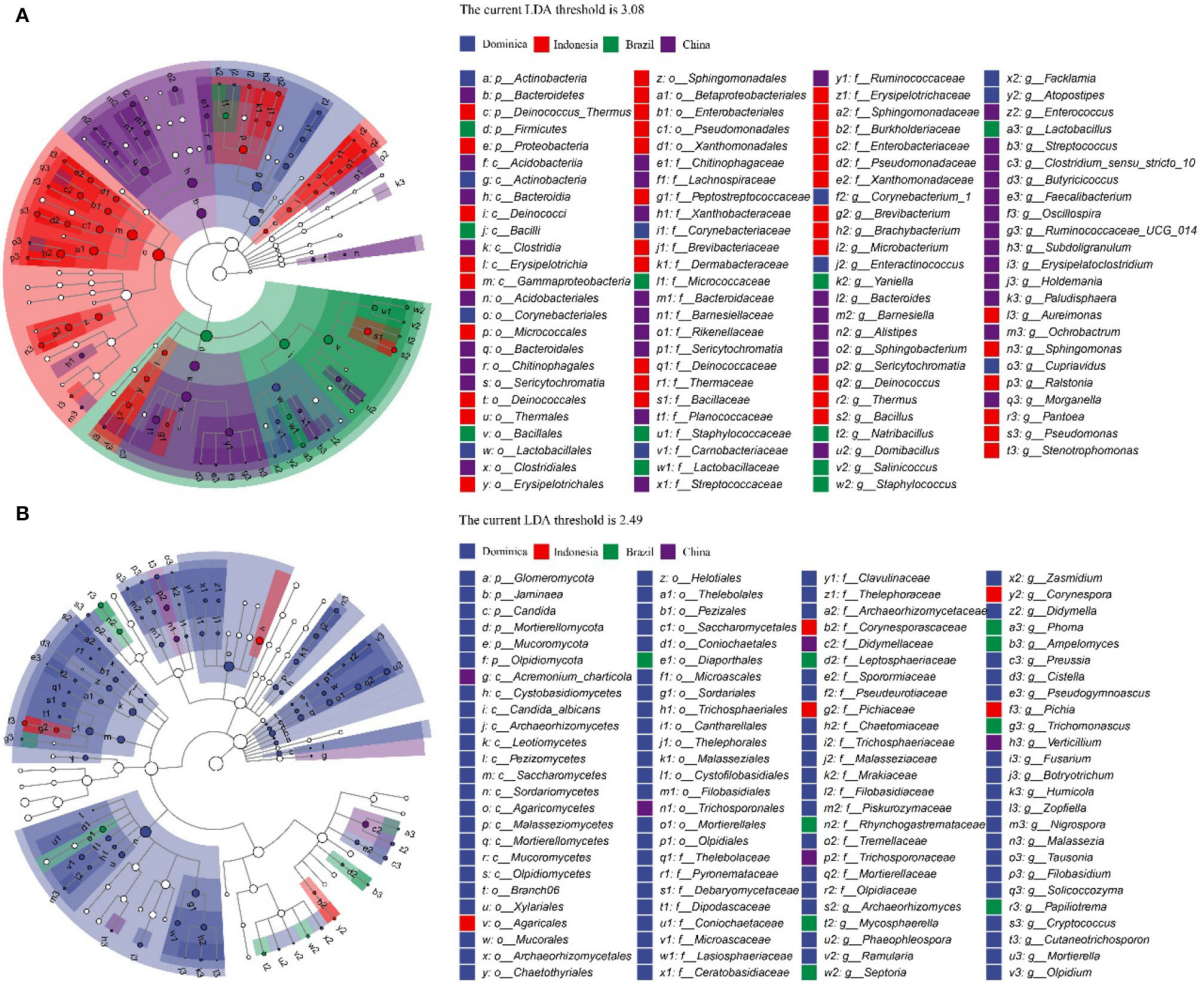
Evolutionary branch map of the bacteria **(A)** and fungus **(B)** with significantly different in cigar tobacco leaves with different treatments.

To explore the different microbiotas among CTLs from different regions, LEfSe analysis was conducted to reveal the significant differences below the level of phylum (Figure 4). The circles from inner to outer represent microbial classification from phylum to genus levels, and corresponding colors in every group denote microbial taxa with a significant difference. Notably, 98 different bacteria appeared in the LDA threshold of 3.08 judging by statistically significant differences (*p* < 0.05), which consist of 5 phyla, 8 classes, 17 orders, 27 families and 41 genera, and 100 different fungi appeared in the LDA threshold of 2.49 judging by statistically significant differences (*p* < 0.05), which consist of 6 phyla, 13 classes, 23 orders, 28 families and 30 genera. Among them, Chinese CTLs had the most specific bacteria, and Dominican CTLs had the most specific fungi.

**Figure 5:**
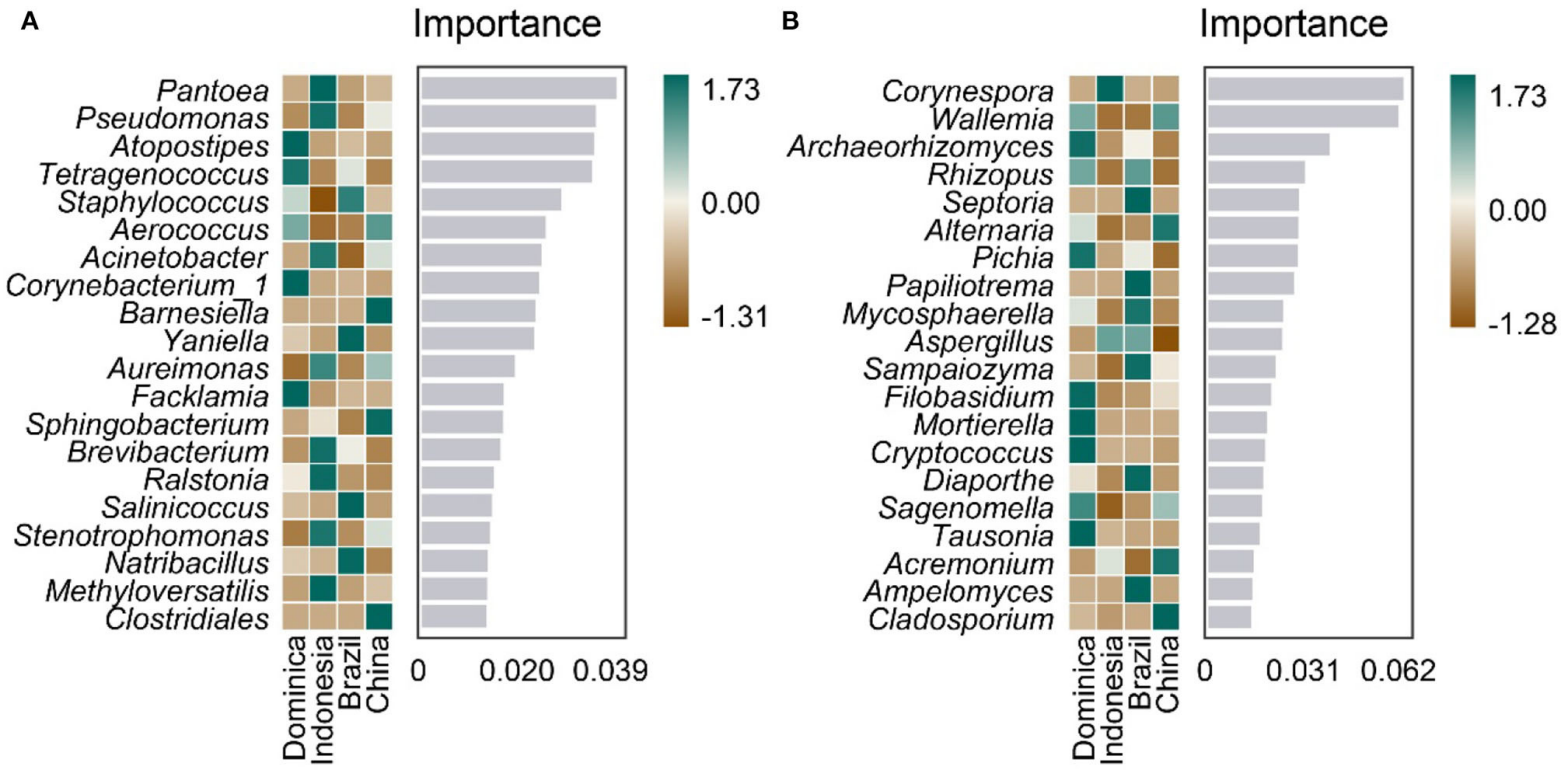
Top 20 identity genus of bacterial **(A)** and fungal **(B)** communities used for discriminating tobacco leaves from different regions.

Furthermore, we analyzed whether microbiotas can be used as biomarkers like other traits, such as flavor, leaf color, and genomic features to differentiate CTLs from different regions. A random forest model was established to distinguish CTLs from different regions using genus-level microbiota. As shown in Figure 5, 20 bacteria and fungi were selected to distinguish CTLs from different regions, bacteria such as *Pantoea, Pseudomonas, Atopostipes, Tetragenococcus*, and Staphylococcus, and fungi such as *Corynespora, Wallemia, Archaeorhizomyces, Rhizopus*, and *Septoria* were of significant importance in distinguishing CTLs from different regions.

### Profiles of the Volatile Flavor Compounds

**Figure 6:**
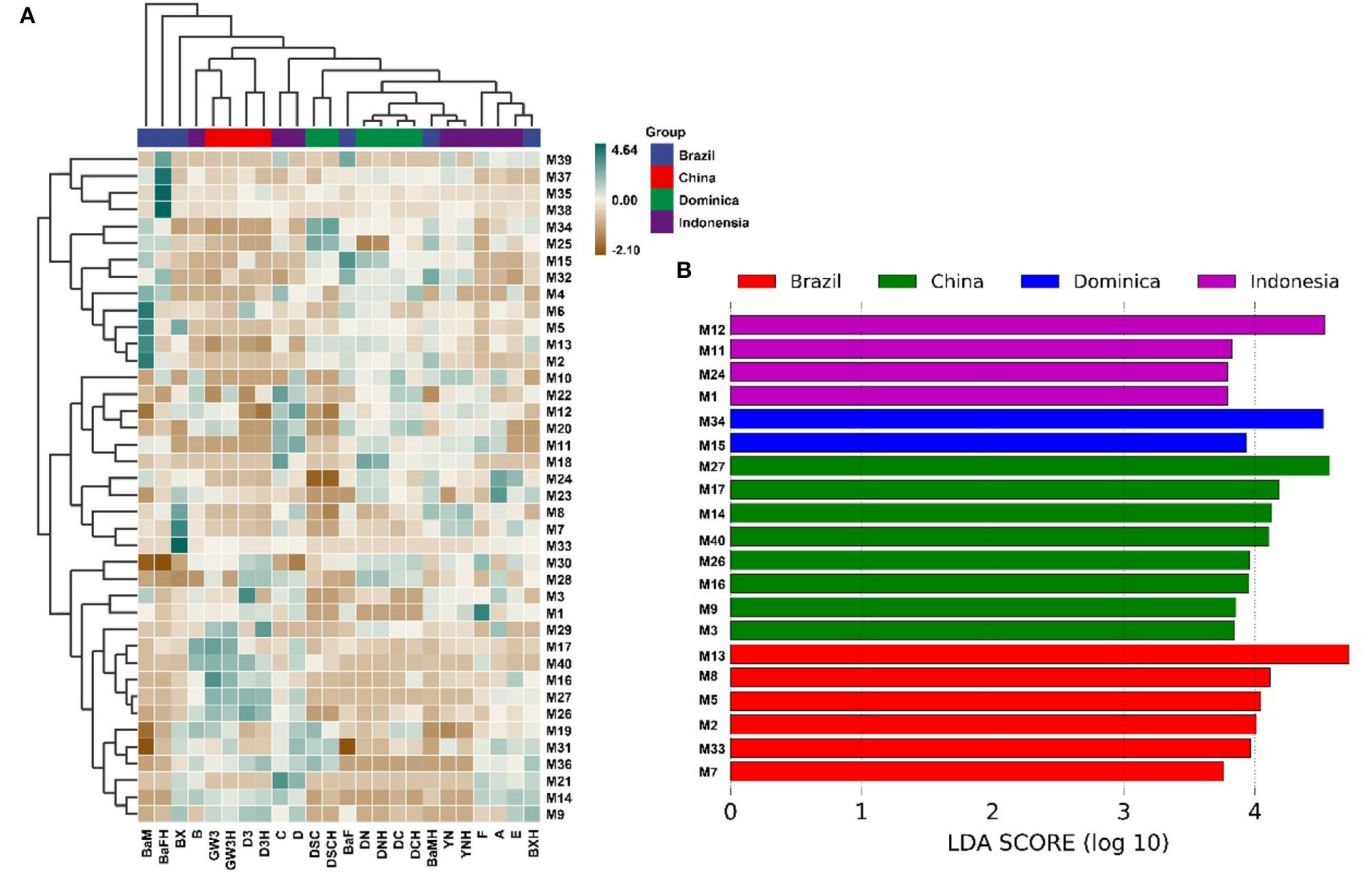
Volatile flavor compounds in the cigar tobacco leaves. Hierarchical clustering of volatile flavor compounds in the cigar tobacco leaves **(A)**, Different volatile flavor compounds in different cigar tobacco leaves **(B)**.

Hundreds of compounds were detected in 24 CTLs by GC–MS analysis, 40 volatile flavor compounds (VFCs) were selected for further analysis and summarized in Table 2, as these compounds were detected with high frequency in all 24 CTLs; meanwhile, these VFCs have been reported to have different aromas that may play an important role in the aroma profile of cigars. Overall, most of these VFCs were aldehydes and ketones. As shown in Figure 6A, VFCs in different CTLs were quite different. Some of the highest content of VCFs were found in Brazilian CTLs. For example, BaM had the highest content of 3-methyl-2-butenal, 3,5,5-trimethyl-2-cyclohexen-1-one, 6-methyl-5-hepten-2-one, and dehydromevalonic lactone, BaFH had the highest content of trimethyl-pyrazine, 2,6-dimethyl-pyrazine, and 2,5-dimethyl-pyridine, and BX had the highest content of octanal. However, some high content of VFCs may produce an offensive odor. For instance, a high concentration of indole has a lasting and robust fecal odor, while highly diluted will produce fragrance (Zeng et al., 2016). LEfSe analysis was used to identify the significantly different VFCs in CTLs from different regions (Figure 6B). Among the 40 VFCs, 20 VFCs appeared in the LDA threshold of 2 judging by statistically significant differences (*p* < 0.05). In detail, 4 VFCs were significantly enriched in Indonesian CTLs, such as (E)-6,10-dimethyl-5,9-undecadien-2-one, 4-(2,6,6-trimethyl-1-cyclohexen-1-yl)-3-buten-2-one, heptanal, and 1H-indole. A total of 2 VFCs were significantly enriched in Dominican CTLs, such as pentanal and 2,4-dimethyl-benzaldehyde. A total of 8 VFCs were significantly enriched in Chinese CTLs, such as benzeneethanol, megastigmatrienone b, benzaldehyde, and β-damascone. A total of 6 VFCs were significantly enriched in Brazilian CTLs, such as 6-methyl-5-hepten-2-one, 6,10-dimethyl-2-undecanoneand, 3-methyl-2-butenal, and 2,5-dimethyl-1H-pyrrole.

**Table 2:** Volatile flavor compounds in cigar tobacco leaves.

**ID**	**Name**	**Aroma description**
M1	1H-Indole	Flowery (Kumar et al., 2018)
M2	2,5-Dimethyl-1H-pyrrole	Sweet, cherry, tobacco (Iqbal et al., 2020)
M3	2,6,6-Trimethyl-2-cyclohexene-1,4-dione	Sour (Gaskett et al., 2005)
M4	2-Methyl-2-butenal	Green, nutty, and fruity (Keyu et al., 2020)
M5	3-Methyl-2-butenal	Flavors of daily and food
M6	3,5,5-Trimethyl-2-cyclohexen-1-one	Earth, increase concentration (Tarantilis and Polissiou, 1997)
M7	(E)-2-Hexenal	Green, fruity, spicy, and cream (Gaunt et al., 1971)
M8	6,10-Dimethyl-2-undecanone	Sweet, nutty
M9	3,7,11,15-Tetramethyl-2-hexadecen-1-ol	Freshness, spicy (Ting et al., 2017)
M10	4,8-Dimethyl-3,7-nonadien-2-one	Fruity
M11	4-(2,6,6-Trimethyl-1-cyclohexen-1-yl)-3-buten-2-one	Sweet, wood, and flowery (Zi-yan et al., 2015)
M12	(E)-6,10-Dimethyl-5,9-undecadien-2-one	Fragrance
M13	6-Methyl-5-hepten-2-one	Smooth, grassy
M14	Benzaldehyde	Cherries, almonds (Kunjapur et al., 2014)
M15	2,4-Dimethyl-benzaldehyde	Mild, sweet, and, and almond (Tahir et al., 2019)
M16	Benzeneacetaldehyde	Hyacinth, fruity, and sweet (Koan Sik et al., 2006)
M17	Benzeneethanol	Sweet, spicy, and nutty (Tian et al., 2020)
M18	Cedrenol	Wood, cream (Bhatia et al., 2008)
M19	Decanal	Sweet, citrus, waxy, and flowery (Gao et al., 2021)
M20	Dehydromevalonic lactone	Sweet (Anisha and Radhakrishnan, 2017)
M21	Farnesol	Sweet, flowery, and green (Muramatsu et al., 2008)
M22	Farnesyl acetone	Sweet, roasted (Villarreal et al., 2015)
M23	2-Pentyl-furan	Bean, fruity, and vegetable (Chung et al., 2020)
M24	Heptanal	Fruity (Van Aardt et al., 2005)
M25	Hexanal	Fruity
M26	Megastigmatrienone a	Flowery, woody (Slaghenaufi et al., 2014)
M27	Megastigmatrienone b	Flowery, woody
M28	Megastigmatrienone c	Flowery, woody
M29	Megastigmatrienone d	Flowery, woody
M30	Neophytadiene	Freshness
M31	Nonanal	Rose, citrus and creamy
M32	Nootkatone	Fruity (Gou et al., 2021)
M33	Octanal	Fruity
M34	Pentanal	Spice raw materials
M35	2,6-Dimethyl-pyrazine	Herbal fragrance (Yan et al., 2021)
M36	Methyl-pyrazine	Sweet
M37	Trimethyl-pyrazine	Sweet
M38	2,5-Dimethyl-pyridine	Earthy, pleasant scent
M39	α-Ionone	Sweet, flowery, and woody (Lalko et al., 2007b)
M40	β-Damascone	Rose, fruity (Lalko et al., 2007a)

### Profiles of Flavor Characteristics of CTLs From Different Regions

**Figure 7:**
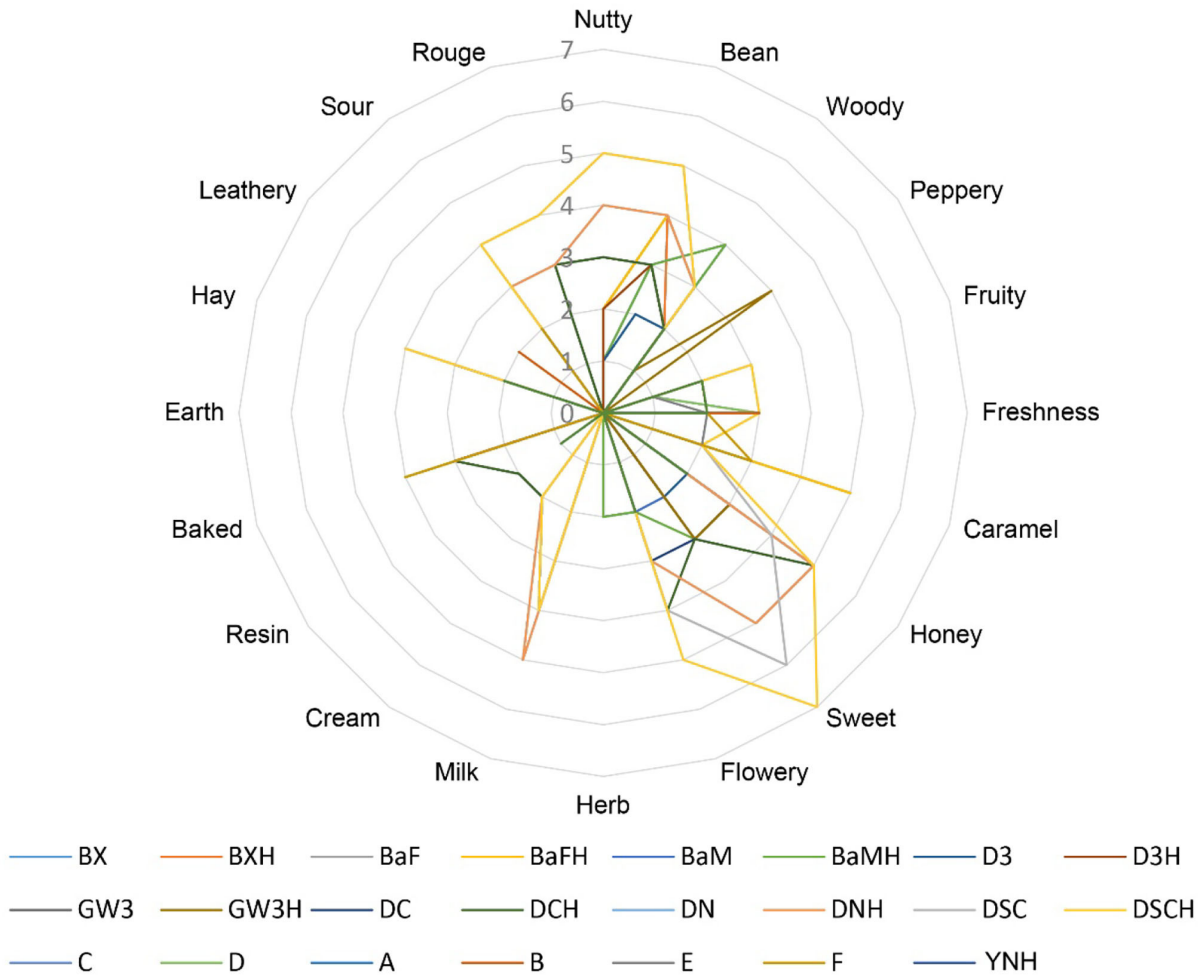
Graph of the sensory score of cigar tobacco leaves.

**Figure 8:**
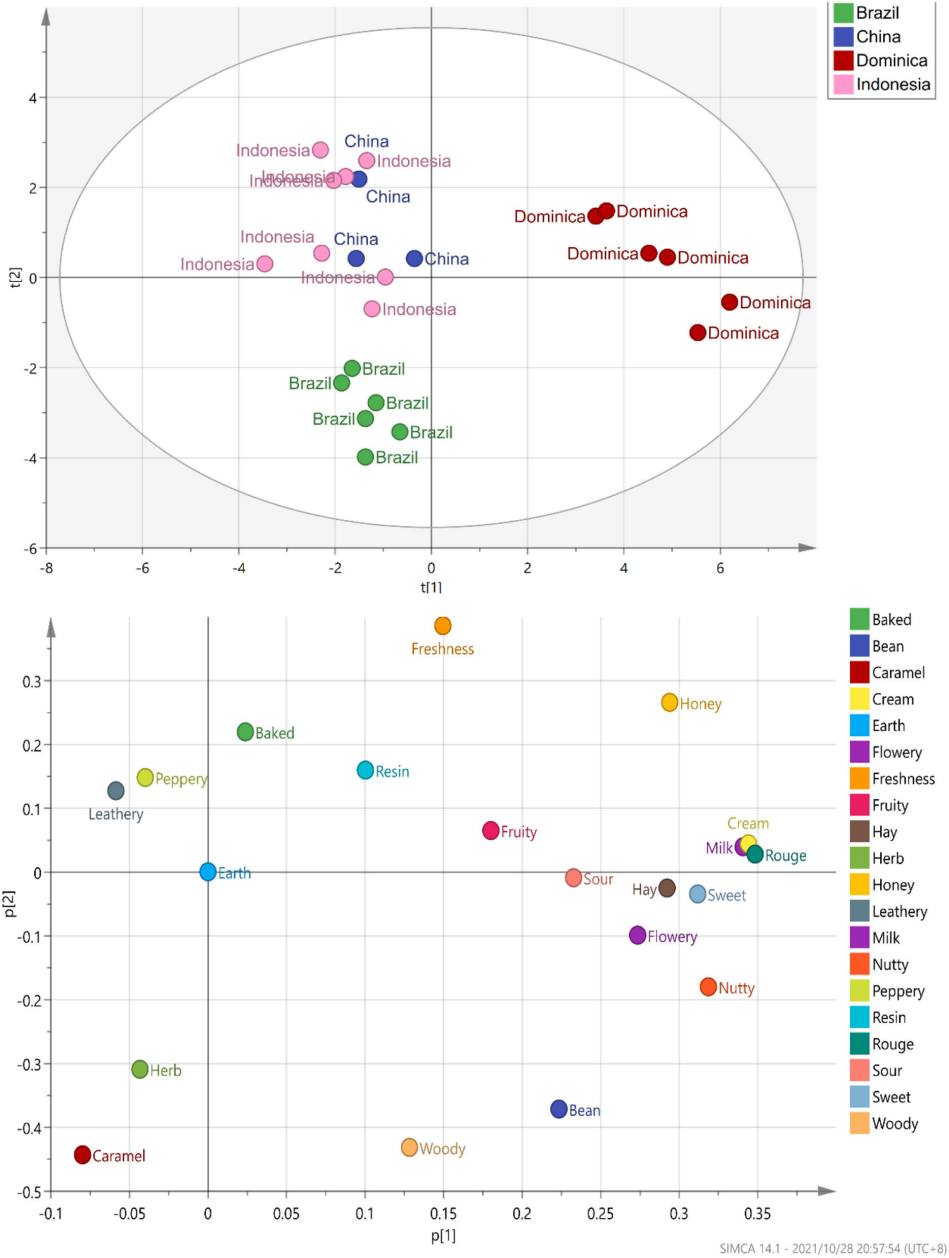
PCA plot marked by sensory analysis score of cigar tobacco leaves.

According to the evaluation criteria of the cigar, 24 CTLs were evaluated and scored. The detailed evaluation score of each sample is shown in Figure 7. Multiple comparison results showed Dominican CTLs had a relatively high honey score, Dominican CTLs DSC and DSCH had the highest scores in nutty and bean, meanwhile, DSCH had the highest sweet score; Brazilian CTLs BaM and BaMH had the highest wood score; Brazilian CTLs BX, BXH, BaF, and BaFH showed a relatively high caramel score. Data of sensory scores of 24 CTLs were subjected to PCA. As shown in Figure 8, CTLs were divided into three clusters according to their flavor characteristics. Cluster 1 (pink and purple color) contained the Indonesian and Chinese CTLs, characterized considerably by leathery, peppery, and baked aroma. Cluster 2 (green color) contained Brazilian CTLs dominated by caramel and herb aroma. Cluster 3 (red color) contained Dominican CTLs with aromas of milk, fruity, sour, cream, flower, nutty, and honey.

### The Contribution of Volatile Flavor Compounds to Flavor Characteristics

**Figure 9:**
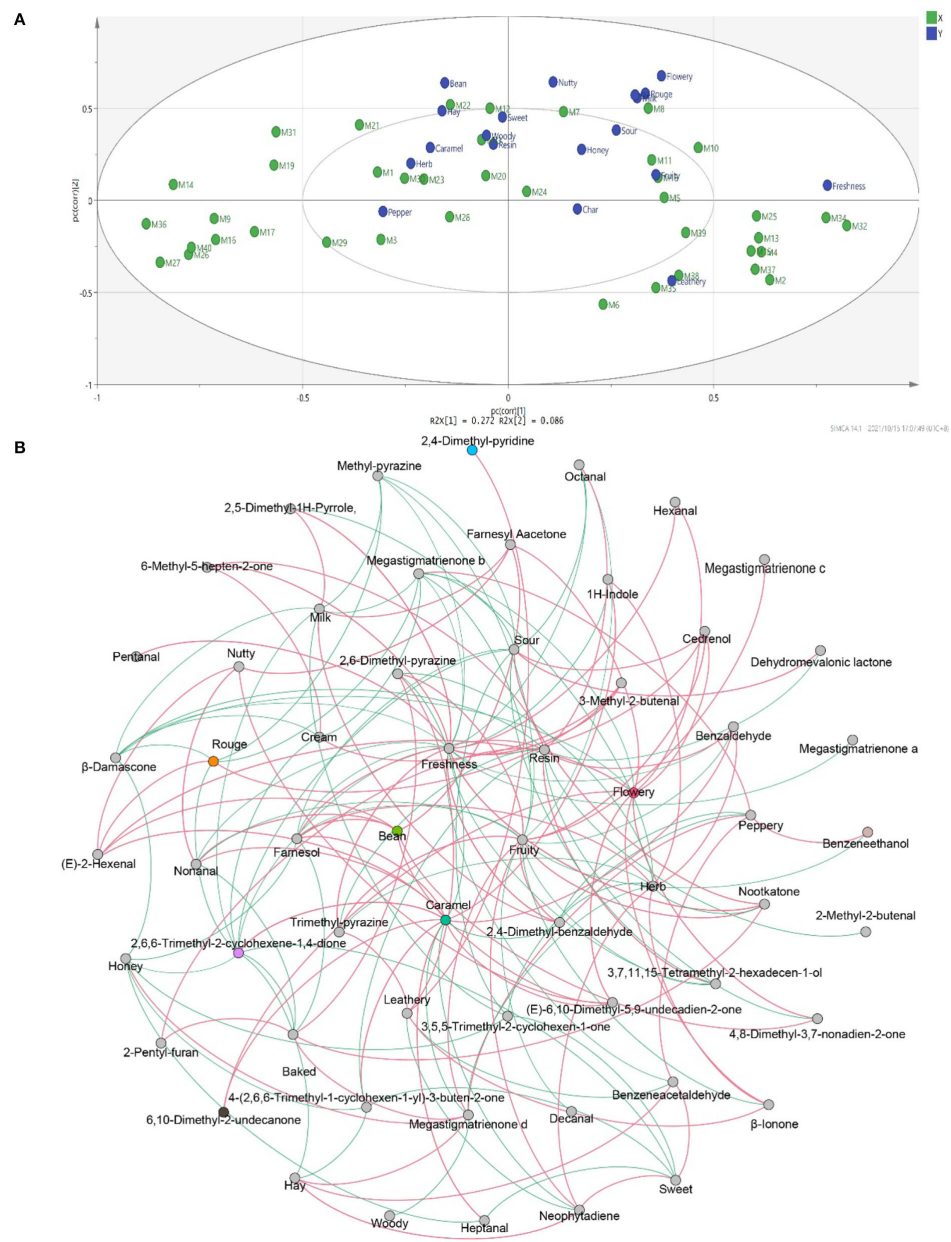
The correlation between flavors and volatile flavor compounds. Correlation loadings plot of PLSR analysis between core volatile flavor compounds and flavors of CTLs **(A)**. Co-occurrence networks of flavors and core volatile flavor compounds. Green line means negative correlation; red line means positive correlation **(B)**.

To study the correlation between flavor characteristics and VFCs of CTLs, PLSR was used to process the data from sensory evaluation and GC–MS. The correlation results indicated that flavor was significantly correlated with some compounds. As shown in Figure 9A, many compounds, such as dehydromevalonic lactone, heptanal, and megastigmatrienone c, lie in the middle of the correlation plot, identified as the essential taste compounds in all of the samples (*p* < 0.05). Nine Y variables, including hay, bean, nutty, flowery, cream, rouge, milk, freshness, and leathery, as well as most volatile compounds, were placed between the inner and outer ellipses, which explain 50 and 100% variances, respectively, indicating that the PLSR model well-explained them. Freshness (*p* < 0.05) positively correlated to nootkatone and pentanal. Leathery positively correlated to 2,6-dimethyl-pyrazine and 2,5-dimethyl-pyridine. It was evident that 6,10-dimethyl-2-undecanone positively correlated to flowery, rouge, and milk. Bean and hay positively correlated to farnesyl acetone, and nutty showed a positive correlation with (E)-2-hexenal. Their relationships were also analyzed by Spearman's rank correlation and visualized by using R software and Gephi (Figure 9B). The same results were found, and other relationships were found. For example, peppery was positively related to benzeneacetaldehyde and benzeneethanol. Fruity was positively related to 2,4–dimethyl–benzaldehyde, cedrenol, megastigmatrienone c, megastigmatrienone d, neophytadiene, nootkatone, 2,6–dimethyl–pyrazine, and trimethyl–pyrazine. Caramel was positively related to 1H–indole, 2,6,6–trimethyl−2–cyclohexene−1,4–dione, 6,10–dimethyl−2–undecanone, 4–(2,6,6–Trimethyl−1–cyclohexen−1–yl)−3–buten−2–one, (E)−6,10–dimethyl−5,9–undecadien−2–one, benzaldehyde, farnesol, heptanal, and nonanal.

### Correlation Analysis of the Microbes and Volatile Flavor Compounds

**Figure 10:**
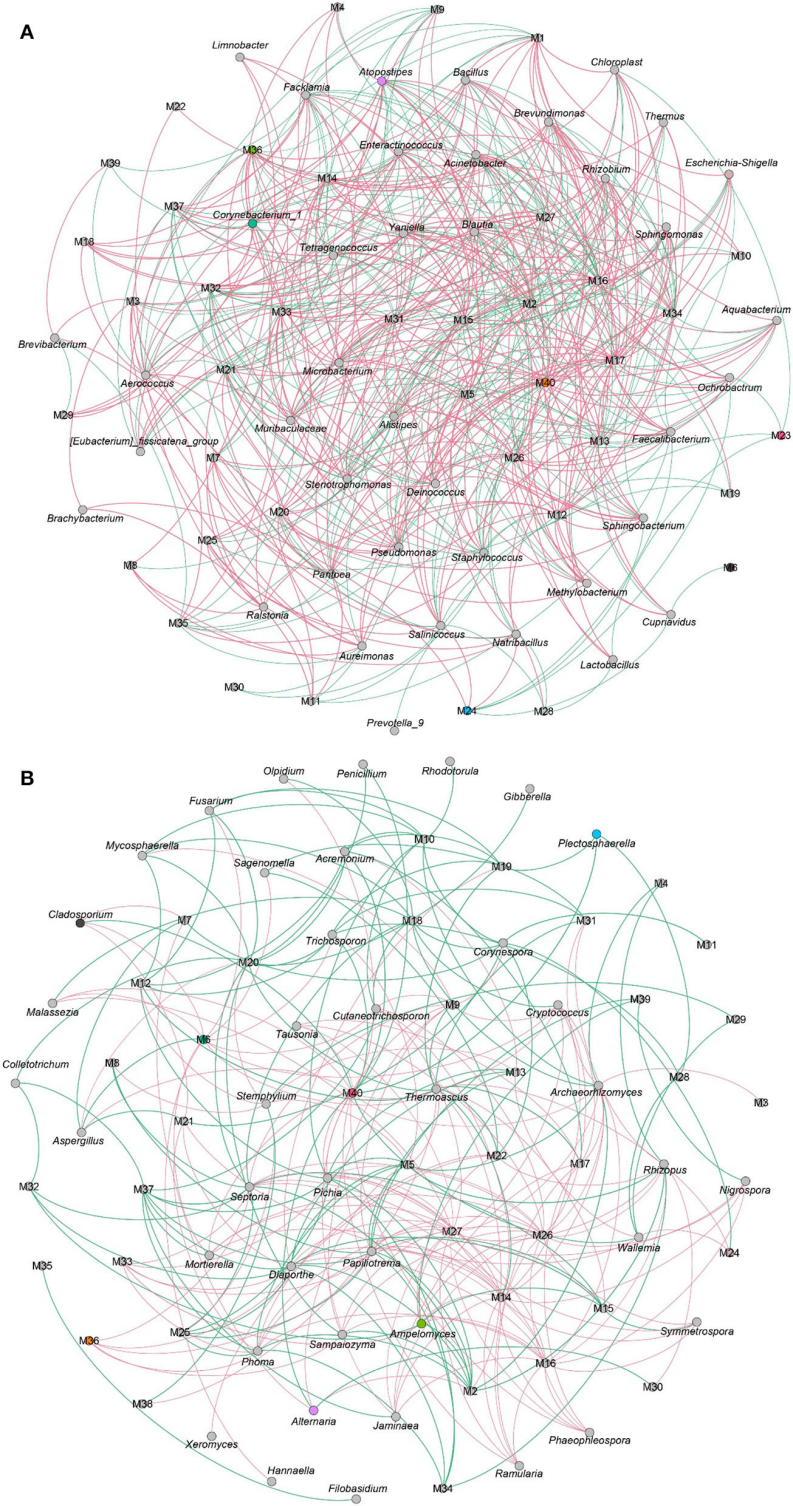
The correlation between microbial community and volatile flavor compounds. Co-occurrence networks of representative bacterial taxa and core volatile flavor compounds. Green line means negative correlation; red line means positive correlation **(A)**. Co-occurrence networks of representative fungal taxa and core volatile flavor compounds. Green line means positive correlation; red line means negative correlation **(B)**.

The VFCs in CTLs not only dependent on raw materials but also related to microbes. As shown in Figure 10A, 3–methyl−2–butenal, (E)−2–hexenal, (E)−6,10–dimethyl−5,9–undecadien−2–one, 2,4–dimethyl–benzaldehyde, cedrenol, farnesol, farnesyl acetone, megastigmatrienone d, neophytadiene, nonanal, nootkatone, trimethyl–pyrazine, and α-Ionone, which were positively correlated with most flavor characteristics, were closely associated with most bacteria. For example, 3–methyl−2–butenal was positively related to *Staphylococcus, Tetragenococcus, Yaniella, Salinicoccus*, and *Papiliotrema*; 2,4–dimethyl–benzaldehyde was positively related to *Staphylococcus, Corynebacterium_1, Tetragenococcus, Yaniella*, and *Enteractinococcus*; nootkatone was positively related to *Corynebacterium_1, Tetragenococcus, Yaniella, Atopostipes, Facklamia*, and *Enteractinococcus*. The connection between fungi and VFCs was less than that of bacteria (Figure 10B), and the number of negative correlations was more than that of positive correlations. For example, benzaldehyde, benzeneacetaldehyde, megastigmatrienone b, and megastigmatrienone c were negatively correlated with all fungi.

## Discussion

As the most widely planted non-food crop in the world, tobacco has distinct regional characteristics. The environment and climate of tobacco planting areas largely determine the quality of CTLs. In addition, the fermentation technology and microbial community endow tobacco leaves with more flavor. This study sought to horizontally characterize the microbial community, VFCs, and flavors of CTLs from four famous cigar producing areas using polyphase detection approaches, revealed the differences in the microbial community, VFCs, and flavors of CTLs from different regions, and investigated the possible relationships between microbes with VFCs and flavors.

The predominant phyla *Firmicutes, Proteobacteria*, and *Actinobacteria* identified in this study were also found in almost all studies associated with the microbiome in tobacco leaves. They are known to be involved in carbon degradation processes such as starch, xylan, and cellulose assimilation. In tobacco fermentation, they act as decomposers to degrade large molecules (cellulose, pectin, and starch) into small molecules (glucose, fructose, and maltose) (Costa et al., 2020). The dominated genera *Staphylococcus, Pseudomonas, Aspergillus, Sampaiozyma*, and *Alternaria* were also reported from time to time in tobacco-related research. However, different tobacco samples have different microbial compositions. Here, according to differences in microbial community abundance, microbial community diversity, and number of specific species, this study demonstrated that CTLs from different regions harbor distinct microbial communities, but the differences in fungal communities are smaller than those in bacterial communities. The previous study has performed baseline cross-sectional characterizations of the microbial communities of cigarillos and packaging materials from different brands and batches, they also have found that different tobacco sample harbors significantly different bacterial microbiotas (Chopyk et al., 2017). The microbial community on CTLs mainly comes from the soil and surrounding environment when growing in the field, and the air when drying (Kandel et al., 2017). The previous studies have concluded that space is an important factor in shaping soil microbial communities at a large spatial scale (Zhang et al., 2020). Therefore, geographic factors are the main force for the construction of the tobacco microbial community. In addition, the microbial communities were also influenced by fermentation methods, fermentation parameters, and fermentation time.

Different microbial communities in CTLs also lead to the difference in VFCs and flavor (Song et al., 2017; Jin et al., 2019; Yang et al., 2021). Among the 40 VFCs, 20 VFCs had significant differences among the four producing areas. In addition, the professional tasters gave different evaluations and scores to different CTLs. Indonesian and Chinese CTLs were characterized considerably by leathery, peppery, and baked aroma. Brazilian CTLs were dominated by caramel and herb aroma. Dominican CTLs had aromas of milk, fruity, sour, cream, flower, nutty, and honey. A similar study has also evaluated the smell of 20 tobacco products using self-defined odor attributes, following quantitative descriptive analysis. The final attribute list as generated by the panel after 14 training sessions consisted of 13 odor descriptors: smoky/burned, vanilla/caramel, coconut, chocolate/cocoa, nutty, raisin, honey, liquorice, hay, red fruit, menthol/mint, tea, and clove. In addition, a four-cluster method was developed to distinguish cherry-flavored, vanilla-flavored, and menthol-flavored products (Krusemann et al., 2019). Supplemented with VFCs, the flavor of CTLs can no longer be objectively described, and different CTLs can be scientifically quantified. A network analysis was conducted to establish the connection between flavor compounds and flavor. Most flavor characteristics are closely related to multiple VFCs. Aldehydes and ketones are the main compounds in these tobacco leaves. The carbonyl groups in the molecular structures of aldehydes and ketones are aroma groups. Most of the compounds with carbonyl groups have a beautiful aroma. For example, β-damascenone has a strong rose and fruity aroma, megastigmatrienone also imparts woody and floral aromas to tobacco leaves. In addition, ester compounds provide tobacco leaves with sweet, fruity, and wine aromas (Xu et al., 2022), alcohol compounds can also enhance the floral and fruity aromas of tobacco leaves (Piornos et al., 2020), pyrazine compounds enhance the nutty and roasted aroma of tobacco leaves (Yan et al., 2021), and furan compounds give tobacco leaves a caramel aroma (Chung et al., 2020). Numerous VFCs constitute the mellow and varied flavor of CTLs. Differences in VFCs in CTLs from regions produce different flavors. Additionally, through the network analysis of microbes and VFCs, it was found that the bacterial community was closely related to most VFCs. The relationships between the fungal community and VFCs were less than the bacterial community, and most of them were negative. On the whole, it may be concluded that the bacterial community had a greater contribution to the flavor of CTLs. Some studies have also reported on their effects on VFCs. For examples, *Staphylococcus* was found to be involved in fat metabolism, and the resulting fatty acids are further degraded to form aromatic compounds (Liu et al., 2020). Our previous studies found that *Acinetobacter, Sphingomonas, Solibacillus*, and *Lysinibacillus*, were the main carbonyl compound-producing microbes in CTLs.

In summary, our results systematically characterized the main characteristics of CTLs from different regions. The microbial communities, VFCs, and flavors of tobacco leaves vary widely due to geographic differences. Furthermore, network analysis revealed the close relationship between microbial community, VFCs, and flavors. These results may help consumers and regulators to increase awareness of CTLs from different regions. For producers, may help regulate and improve the cultivation, fermentation, and production of the cigar.

## Data Availability Statement

The datasets presented in this study can be found in online repositories. The names of the repository/repositories and accession number(s) can be found in the article/supplementary material.

## Author Contributions

TZ: conceptualization, data curation, formal analysis, methodology, software, and writing—original drafting. QZ and YL: investigation, methodology, and resources. ZY, XW, and PL: methodology, resources, and project administration. JZ, GD, and DL: funding acquisition, supervision, and writing—reviewing and editing. All authors have read and agreed to the published version of the manuscript.

## Funding

This work was supported by the National Key Research and Development Program of China (2019YFC1605800), the China National Tobacco Corporation 2020 Major Science and Technology Project 110202001040(XJ-02), and the Major projects on constructing the mellow and sweet fragrance styles of Chinese-Stylistic Tobacco (ctx201905).

## Conflict of Interest

The authors declare that this study received funding from China Tobacco Sichuan Industrial Co., Ltd. The funder had the following involvement in the study: study design, data collection and analysis, decision to publish, or preparation of the manuscript. QZ, YL, PL, ZY, and DL were employed by China Tobacco Sichuan Industrial Co., Ltd. The remaining authors declare that the research was conducted in the absence of any commercial or financial relationships that could be construed as a potential conflict of interest.

## Publisher's Note

All claims expressed in this article are solely those of the authors and do not necessarily represent those of their affiliated organizations, or those of the publisher, the editors and the reviewers. Any product that may be evaluated in this article, or claim that may be made by its manufacturer, is not guaranteed or endorsed by the publisher.
